# Improvement of the clinical applicability of the Genomic Grade Index through a qRT-PCR test performed on frozen and formalin-fixed paraffin-embedded tissues

**DOI:** 10.1186/1471-2164-10-424

**Published:** 2009-09-10

**Authors:** Jérôme Toussaint, Anieta M Sieuwerts, Benjamin Haibe-Kains, Christine Desmedt, Ghizlane Rouas, Adrian L Harris, Denis Larsimont, Martine Piccart, John A Foekens, Virginie Durbecq, Christos Sotiriou

**Affiliations:** 1 Translational Research Unit, Jules Bordet Institute, Brussels, Belgium; 2 Department of Medical Oncology, Erasmus Medical Center - Josephine Nefkens Institute and Cancer Genomics Centre, Rotterdam, The Netherlands; 3 Molecular Oncology Laboratories, John Radcliffe Hospital, Oxford, United Kingdom

## Abstract

**Background:**

Proliferation and tumor differentiation captured by the genomic grade index (GGI) are important prognostic indicators in breast cancer (BC) especially for the estrogen receptor positive (ER+) disease. The aims of this study were to convert this microarray index to a qRT-PCR assay (PCR-GGI), which could be realized on formalin fixed paraffin embedded samples (FFPE), and to assess its prognostic performance and predictive value of clinical benefit in early and advanced ER+ BC patients treated with tamoxifen.

**Methods:**

The accuracy and concordance of the PCR-GGI with the GGI was assessed using BC patients for which frozen and FFPE tissues as well as microarray data were available (n = 19). The evaluation of the prognostic value of the PCR-GGI was assessed on FFPE material using a consecutive series of 212 systemically treated early BC patients. The predictive performance for tamoxifen benefit was assessed using two ER+ BC populations treated either with adjuvant tamoxifen only (n = 77+139) or first-line tamoxifen for advanced disease (n = 270).

**Results:**

The PCR-GGI is based on the expression of 8 genes (4 representative of the GGI and 4 reference genes). A significant correlation was observed between the microarray-derived GGI and the qRT-PCR assay using frozen (ρ = 0.95, p < 10E-06) and FFPE material (ρ = 0.89, p < 10E-06). The prognostic performance of the PCR-GGI was confirmed on FFPE samples (HR_univar. _= 1.89; [95CI:1.01-3.54], p = 0.05). The PCR-GGI further identified two subgroups of patients with statistically different time to distant metastasis free survival (DMFS) across the two cohorts of ER+ BC patients treated with adjuvant tamoxifen. Additionally, the PCR-GGI was associated with response to tamoxifen in the advanced setting (HR_univar. _= 1.98; [95CI:1.51-2.59], p = 6.9E-07).

**Conclusion:**

PCR-GGI recapitulates in an accurate and reproducible manner the performances of the GGI using frozen and FFPE samples.

## Background

Gene expression profiling has resulted in paradigm shift in the way that researchers and clinical investigators view breast cancer (BC) biology. During the last years, several groups have evaluated the potential of gene expression profiles to improve BC prognostication. This has resulted in the identification of several gene signatures, most of them outperforming current clinico-pathological parameters in predicting clinical outcome (reviewed in [1]).

One example is the genomic grade index (GGI) developed by our group [2]. The hypothesis beyond the development of this index was to improve BC grading and its prognostic value. This index includes 97 genes that are consistently differentially expressed between low and high histological grade breast tumors. Of interest, the majority of them are related to cell cycle and proliferation. One of the major findings of that study was that GGI could assign intermediate histological grade tumors into two distinct subgroups whose gene expression profiles and clinical outcome was similar to the ones of low and high histological grade tumors respectively. In addition, GGI could identify two clinically relevant ER+ subtypes with very distinct clinical outcomes in both systemically untreated and tamoxifen only treated BC patients [3].

Finally, comparative studies of the GGI with other prognostic gene signatures in the context of a large meta-analysis involving ~3000 BC patients suggest that most of these signatures have a similar prognostic performance which is limited to the ER+ disease, and that the proliferation and cell-cycle genes represent the driving force of these signatures [4,5].

In this study we aimed to transpose the GGI onto a qRT-PCR assay based on a minimal set of genes that could recapitulate in an accurate and reproducible manner the prognostic performance of GGI using both frozen and paraffin-embedded (FFPE) tumor samples, to facilitate its use in clinical practice. Furthermore, we investigated the performance of this assay to predict benefit to adjuvant tamoxifen in early BC patients or to first line tamoxifen in advanced BC patients.

## Results

### Development of the reduced genomic qRT-PCR grade index (PCR-GGI)

The first step in the development of the PCR-GGI was to identify a minimum number of genes that could recapitulate the performance of the original GGI.

To this end, we first tested the correlation of the expression values of several random combinations of minimum 4 genes, covering all phases of the cell cycle, with the original GGI. This correlation was performed using gene expression data from two publicly available microarray data sets namely the NKI [6] and VDX [7]. We selected a set of four genes (*MYBL2, KPNA2, CDC2 *and *CDC20*) based on their high significant correlation with the original GGI (0.95 and 0.94 (all *P *< 10^-6^) in the NKI and VDX data sets respectively). We also selected 4 reference genes (GUS, TBP, RPLPO and TFRC). Although all housekeeping genes gave similar results for normalizing the data, the variance of the estimation of the housekeeping Ct value was reduced by using the mean of these 4 reference genes (data not shown).

The prognostic performance of this reduced gene set (GGIreduced) was then compared to the performance of the original GGI in the NKI and VDX data sets. This comparison revealed similar prognostic performances (NKI data set: HR_GGI _= 1.73; [95%CI: 1.40-2.14], p = 4.2E-07 and HR_GGIreduced _= 1.49; [95%CI: 1.21-1.82], p = 1.2E-04; VDX data set: HR_GGI _= 1.41; [95%CI: 1.13-1.76], p = 2.0E-03 and HR_GGreduced _= 1.36; [95%CI:1.09-1.68], p = 5.8E-03). Of interest, the correlation values with the original GGI and the prognostic performances were not improved when additional genes were taken into consideration (data not shown).

The second step in developing the PCR-GGI was to convert these four genes into a qRT-PCR index. To this end, we designed primers for the four selected genes as well as for four reference genes considering the SYBR-based real-time PCR assay platform. We assessed the concordance between the microarray-derived GGI and the PCR-GGI measured on frozen and FFPE tissues using a series of 19 primary BC from which frozen, FFPE tissues and their corresponding microarray data were available, further referred as "IJBtest". A statistically significant correlation was observed between the expression levels of the microarray-derived GGI and the PCR-GGI assay using frozen samples (ρ = 0.95 [95%CI: 0.86-0.98], p = 3.6E-09) as well as FFPE samples (ρ = 0.89 [95%CI: 0.72-0.96], p = 8.26E-07) (see Table 1). Similarly, a statistically significant correlation was found between the expression levels of the PCR-GGI derived from frozen and FFPE samples respectively (ρ = 0.85 [95%CI: 0.64-0.94], p = 7.7E-06).

**Table 1:** Correlation between the original GGI index and the qRT-PCR index derived from frozen and FFPE samples.

**genes**	**GGI vs GG qRT-PCR** **(*Frozen*)**			**GGI vs GG qRT-PCR** **(*FFPE*)**			**GG qRT-PCR** ***Frozen *vs *FFPE***		
	**Cor.coef**	**CI95%**	**p.val.**	**Cor.coef**	**CI95%**	**p.val.**	**Cor.coef**	**CI95%**	**p.val.**
*CDC20*	0.933	[0.825-0.975]	1.70E-08	0.732	[0.403-0.893]	5.55E-04	0.775	[0.482-0.912]	1.60E-04
*CDC2*	0.644	[0.253-0.854]	3.93E-03	0.819	[0.57-0.93]	3.22E-05	0.694	[0.336-0.877]	1.39E-03
*MYBL2*	0.772	[0.478-0.911]	1.73E-04	0.589	[0.168-0.828]	4.92E-05	0.831	[0.596-0.935]	1.91E-05
*KPNA2*	0.762	[0.458-0.906]	2.37E-04	0.64	[0.247-0.852]	4.22E-03	0.73	[0.4-0.893]	5.78E-04
*4 genes*	0.95	[0.86-0.98]	3.6E-09	0.89	[0.72-0.96]	8.26E-07	0.85	[0.64-0.94]	7.7E-06

Interestingly, the correlations between the microarray-derived GGI and the PCR-GGI assay on frozen and FFPE samples were higher when the 4 genes were combined together compared to the individual genes (see table 1).

### Prognostic performance of the PCR-GGI

To evaluate the prognostic performance of the PCR-GGI, we considered an independent population of 212 primary BC FFPE samples originated from patients consecutively diagnosed at the Jules Bordet Institute between 1995 and 1996 (referred as IJB95/96). The clinico-pathological characteristics are summarized in Table 2.

**Table 2:** Population characteristics

	***OXFD***	***IJB95/96***	***JNIadj***	***JNImeta***
	*(n = 77)*	*(n = 212)*	*(n = 139)*	*(n = 270)*
Mean age at diagnosis				
(years)	**64**	**58.5**	**64**	**58**
(range)	**(40-86)**	**(31-87)**	**(46-87)**	**(26-89)**

Menopausal status				
Premenopausal	**/**	**54**	**7**	**95**
Postmenopausal	**/**	**135**	**132**	**175**
UK*	**77**	**23**	**0**	**0**

Event free survival	*DMFS/*	*DMFS/*	*RFS/*	*Progression/*
(mean; months)	**67**	**78.7**	**46**	**13.4**
(range)	**(0.26-129)**	**(0.13-142.27)**	**(2-129)**	**(1-70)**

Death				
Yes	**/**	**24**	**51**	**179**
No	**/**	**188**	**88**	**91**

Tumor size				
≤2	**31**	**95**	**25**	**/**
>2	**46**	**95**	**81**	
(mean)	**3.14**	**2.29**	**2.6**	
(range)	**(1-7)**	**(0.15-8)**	**(1-8)**	
UK*		**22**	**33**	

Histological grade				
1	**13 (16.7%)**	**37 (17.5%)**	**1 (0.01%)**	**1 (0.003%)**
2	**40 (51.2%)**	**89 (42.5%)**	**16 (11.5%)**	**34 (12.6%)**
3	**13 (16.7%)**	**83 (39.2%)**	**77 (55.4%)**	**154 (57.0%)**
UK*	**11 (15.4%)**	**3 (0.01%)**	**45 (32.3%)**	**81 (30.0%)**

Number of metastasis sites				
0	**50**	**171**	**/**	**/**
1	**24**	**20**		
2	**3**	**19**		
≥ 3	**0**	**2**		

Histo. Estrogen Receptor status (>10% or ≥10 fmol/mg^$^)				
Positive	**77**	**114**	**139^$^**	**270^$^**
Negative	**0**	**62**	**0^$^**	**0^$^**
UK*	**0**	**36**	**0^$^**	**0^$^**

Histo. Progesterone Receptor status (>10% or = 10 fmol/mg)				
Positive	**/**	**82**	**88^$^**	**216^$^**
Negative		**90**	**43^$^**	**46^$^**
UK*		**40**	**8^$^**	**8^$^**

Histo. Ki-67 status (>15%)				
Positive	**/**	**64**	**/**	**/**
Negative		**98**		
UK*		**50**		

No Positive Lymph Nodes (at surgery)				
0	**45**	**115**	**0**	**121**
1 - 3	**28**	**49**	**96**	**117**
≥ 4	**0**	**36**	**43**	**28**
UK*	**4**	**12**	**0**	**4**

*:UK = Unknown.

**Figure 1:**
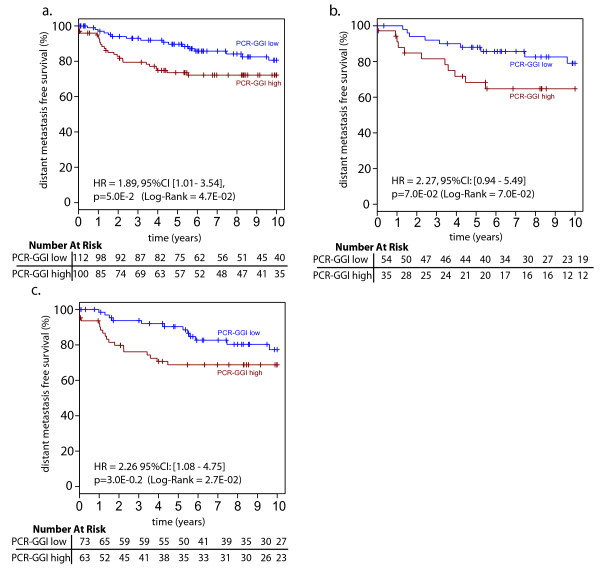
**IJB95/96 FFPE Population**: Distant metastasis free survival analysis for the IJB95/96 FFPE population by qRT-PCR grading. **(A) **Analysis of the whole population: PCR-GGI low = blue and PCR-GGI high = red **(B) **Analysis of the histological grade 2 (HG2) tumors by qRT-PCR grading. The 89 patients with HG2 tumors were separated into low-and high-risk subsets by this signature as PCR-GGI low = blue and PCR-GGI high = red. **(C) **Analysis of the ER+ samples: PCR-GGI low = blue and PCR-GGI high = red. Difference in relapse-free survival between two groups is summarized by the hazard ratio (HR) for recurrence with its 95% CI. All statistical tests were two-sided.

The prognostic performances of the individual four genes (included in the PCR-GGI) were compared to the one of the PCR-GGI and summarized in Table 3. The group of patients with high PCR-GGI scores was associated with a higher risk of recurrence than the group of patients with low PCR-GGI (HR = 1.89; [95%CI: 1.01-3.54], p = 0.05; see Figure 1a). However, this association was not significant but showed a trend towards significance in the multivariate analysis. Indeed, when selecting the age of the patient, the size of the tumor, the centrally reviewed histological grade, the nodal status and the PCR-GGI as variables for the multivariate analysis using a backward stepwise selection for the identification of the covariates to be kept in the final Cox regression model for distant metastasis free survival (DMFS), only the nodal status (HR = 3.10; [95%CI: 1.53-6.27], p = 0.002) and PCR-GGI (HR = 1.89; [95%CI: 0.94-3.77], p = 0.07) were still present in the last step of the analysis.

**Table 3:** Prognostic performance of the individual 4 genes included in the PCR-GGI

	**HR**	**CI (95%)**	**P.val**
	**HR**	**CI (95%)**	**P.val**
*CDC20*	1.14	0.98-1.14	0.096
*MYBL2*	1.07	0.94-1.22	0.304
*KPNA2*	1.12	1.04-1.21	0.004
*PCR-GGI*	1.89	1.01-3.54	0.05

As expected, histological grade 2 tumors (n = 89) spanned the expression levels of PCR-GGI of histological grade 1 and 3 (data not shown). Similarly to GGI, PCR-GGI divided histological grade 2 tumors into two subgroups with distinct clinical outcome although of borderline significance (HR = 2.27; [95%CI: 0.94-5.49], p = 0.07; see Figure 1b).

Since we previously demonstrated that the GGI was particularly performant at identifying high and low risk patients within the ER-positive population [3], we restricted our analysis to the ER+ subpopulation of the "IJB95/96" cohort. In order to identify these ER-positive patients, we evaluated ER by qRT-PCR and defined a cut-off for positivity based on the binomial repartition of ER score (see Material and methods). Sixty-four percent (136/212) of the samples were considered as ER-positive. Overall concordance with IHC data assessed on 162 FFPE samples (76%) for which both IHC and qRT-PCR results were available was of 75% with a correlation of 0.38 (p = 9E-07). When PCR-GGI was applied to these ER+ samples, Kaplan-Meier survival curves and Cox analyses revealed a significant association between a high risk of recurrence and a high PCR-GGI score (HR = 2.26; [95%CI: 1.08-4.75], p = 0.03; see Figure 1c).

Several studies have demonstrated that the widely used proliferation marker Ki-67 evaluated by IHC predicts clinical outcome [8]. We therefore evaluated the prognostic value of Ki-67 by IHC in the IJB95/96 series (N = 160). There was no significant association between Ki-67 and the risk of recurrence both when considering Ki-67 as a continuous variable (HR = 1.01; [95%CI: 0.99-1.03], p = 0.19) or as a binary variable using the cut-off of >15%;(HR = 1.03; [95%CI: 0.51-2.07], p = 0.93).

### Assessment of the performance of the PCR-GGI in tamoxifen-treated BC patients

In order to assess a potential predictive value of the PCR-GGI for clinical benefit to tamoxifen, we evaluated its performance in three independent ER-positive BC populations treated either with adjuvant tamoxifen only or with first-line tamoxifen for advanced disease.

#### i) ER-positive BC patients treated with adjuvant tamoxifen only

The potential value of the PCR-GGI to predict DMFS in tamoxifen-treated patients was first evaluated in a patient's series on which the original GGI was previously computed [3] (n = 77), offering the possibility to compare the performance of GGI and PCR-GGI (referred as OXFD, see Table 2). Of note, 62% (45/73) of the samples originated from node-negative patients.

**Figure 2:**
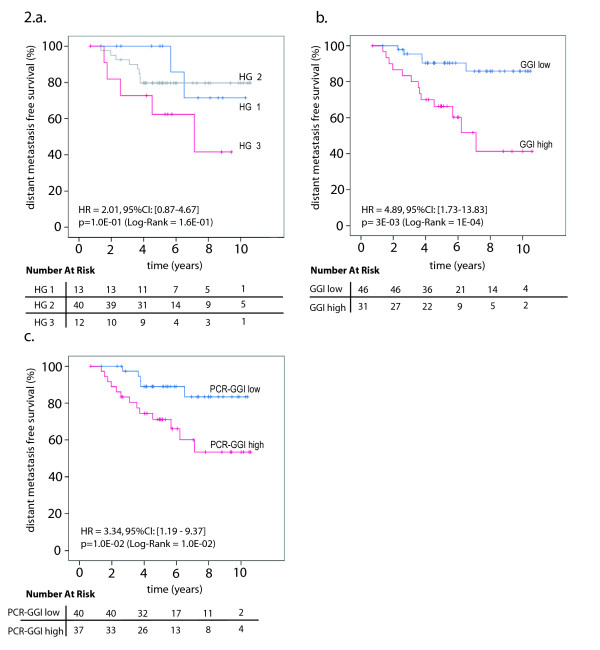
**OXFD Population**: Distant metastasis free survival analysis for the Oxford (OXFD) ER+ frozen population. **(A) **Analysis of the whole population by histological grade (HG1 (blue), HG2 (gray) and HG3 (red)). **(B) **Analysis of the whole population by gene expression grade index (GGI) (GGI low = blue and GGI high = red). **(C) **Analysis of the whole population by qRT-PCR grading: PCR-GGI low = blue and PCR-GGI high = red. Difference in relapse-free survival between two groups is summarized by the hazard ration (HR) for recurrence with its 95% CI. All statistical tests were two-sided.

We observed a significant association between a high PCR-GGI score and a higher risk of recurrence (HR = 3.34; [95%CI: 1.19-9.37], p = 0.01). This performance appeared similar to the one of the original GGI (HR_univar. _= 4.89; [95%CI: 1.73-13.83]; p = 0.003) and superior to the one of the histological grade (HR_univar. _= 2.01; 95%CI: [0.87-4.67]; p = 0.10). The Kaplan-Meier curves for the histological grade, the PCR-GGI and the original GGI are illustrated in Figure 2. When selecting the age of the patient, the size of the tumor, the histological grade, the nodal status and the PCR-GGI as variables for the multivariate analysis using a backward stepwise selection for the identification of the covariates to be kept in the final Cox regression model for DMFS, only the tumor size (HR = 6.22; [95%CI: 1.35-28.75], p = 0.02), the age of the patient (HR = 0.27; [95%CI: 0.07-1.03], p = 0.06), and PCR-GGI (HR = 2.53; [95%CI: 0.80-7.99], p = 0.12) were still present in the last step of the analysis.

**Figure 3:**
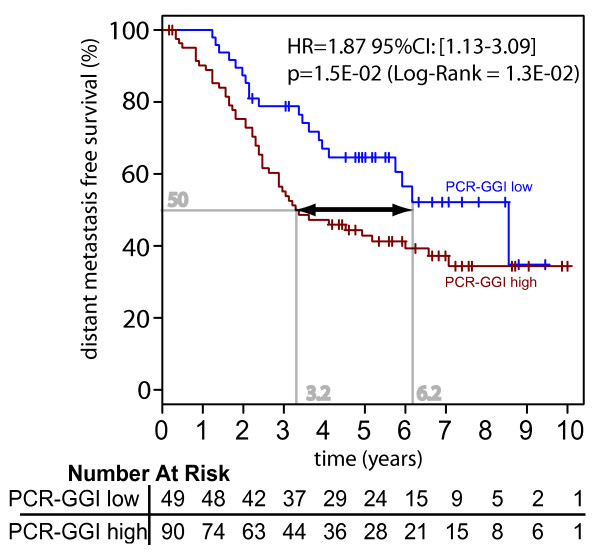
**JNIadj Population**: Relapse free survival analysis for JNIadj ER+ node positive tamoxifen only treated population by qRT-PCR grading: PCR-GGI low = blue and PCR-GGI high = red. The low-risk patients recurring 3 years later compared to high-risk patients (difference observed at 50% survival). Difference in relapse-free survival between two groups is summarized by the hazard ratio (HR) for recurrence with its 95% CI. All statistical tests were two-sided.

The PCR-GGI was further applied to an independent population of 139 ER-positive BC samples originated from node positive patients that received adjuvant tamoxifen for primary disease (referred as JNIadj; see Table 2). Again, we observed a statistically significant association between a high PCR-GGI score and a higher risk of recurrence (HR = 1.87; [95%CI: 1.13-3.09], p = 0.015). The Kaplan-Meier curves are illustrated in Figure 3. Interestingly, a 3-years delay of recurrence was observed between the patients with low and high PCR-GGI.

The PCR-GGI remained significantly associated with DMFS in the multivariate analysis (HR = 1.97; [95%CI: 1.09-3.55], p = 0.024) together with tumor size (HR = 2.65; [95%CI: 1.25-5.61], p = 0.011), but not with the age of the patient. Of note, the nodal status and the histological grade were not included in the analysis due to the node-positivity of all patients and the high number of missing values for the histological grade.

#### ii) ER+ BC patients treated with first-line tamoxifen for metastatic disease (JNImeta)

The PCR-GGI was applied to an independent population of 270 ER+ BC samples originated from patients that received first-line tamoxifen for advanced disease (referred as JNImeta; see Table 2).

**Figure 4:**
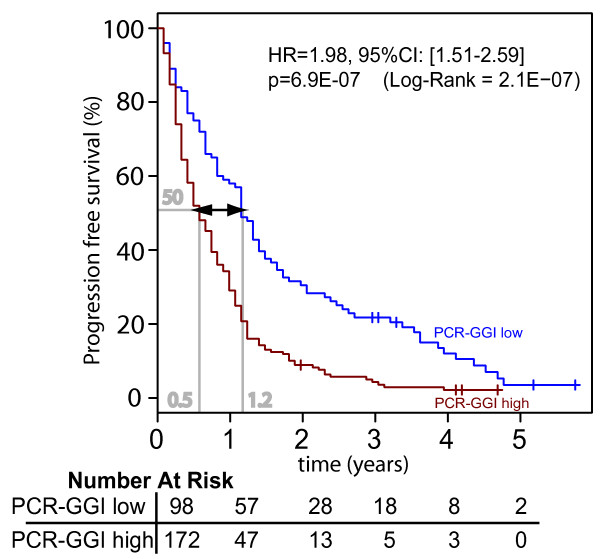
**JNImeta Population**: Progression free survival (PFS) analyses for JNImeta ER+ advanced BC tamoxifen only treated patients by qRT-PCR grading: PCR-GGI low = blue and PCR-GGI high = red. The low-risk patients recurring 7.5 months later compared to high-risk patients (difference observed at 50% survival). Difference in progression-free survival between two groups is summarized by the hazard ratio (HR) for recurrence with its 95% CI. All statistical tests were two-sided.

A statistically significant association was observed between a high PCR-GGI score and progression-free survival (PFS) (HR = 1.98; [95CI: 1.51-2.59], p = 6.9E-07); disease progression after start of first-line tamoxifen being observed with a delay of 7.5 months in the patients with low PCR-GGI compared to the patients with high PCR-GGI at 50% PFS (see Figure 4). The PCR-GGI remained significantly associated with PFS in the multivariate analysis (HR = 1.98; [95CI: 1.51-2.59], p = 6.9E-07). We also observed a significant association between the PCR-GGI and response to tamoxifen as the patients with a low PCR-GGI score had a higher probability of response to tamoxifen (74.5% versus 53% for the patients with high PCR-GGI scores, p = 5E-04). The PCR-GGI correctly classified 81 of the 106 non-responders patients in the high grade subgroup of samples (76.5% sensitivity, 95%CI: 67-84%) and 73 of the 164 patients with objective response (OR) or stable disease lasting over six months in the low grade subgroup of samples (44.5% specificity, 95%CI: 37-52%) with an odds ratio of 2.59 (95%CI: 1.51-4.48).

## Discussion

In this study, we have developed a qRT-PCR grade index (PCR-GGI) composed of four genes associated with cell cycle progression and proliferation initially included in the GGI [2]. This gene set includes *MYBL2, KPNA2, CDC2 *and *CDC20*, which together cover all phases of the cell cycle. Indeed, *MYBL2 *encodes a nuclear protein involved in cell cycle progression; *KPNA2 *is involved in the import of proteins to the nuclear envelope and acts as a regulator of cell cycle checkpoint mediators; *CDC2 *encodes a catalytic subunit of the highly conserved protein kinase complex known as M-phase promoting factor (MPF) which is essential for G1/S and G2/M phase transitions; and, *CDC20 *encodes a protein acting as a regulatory protein interacting with several other proteins at multiple points in the cell cycle.

We demonstrated that a quantitative assessment of a very small number of genes is sufficient to recapitulate the performance of the original GGI, especially in ER-positive breast cancer. This could be explained by the fact that the expression levels of most genes of the GGI signature are highly correlated with one another. Two out of the four genes included in our PCR-GGI signature were also present in a number of previously reported prognostic signatures; *MYBL2 *was part of the Recurrence Score developed by Paik et al. [9] as well as the microarray signatures defined by Naderi et al. [10] and Miller et al. [11], whereas the *KPNA2 *gene was present in the 76-gene signature of Wang et al. [7], which has been applied successfully on a cohort of node-negative estrogen receptor positive breast cancer patients to predict benefit of tamoxifen [12].

We further showed that the averaged expression of the four selected genes evaluated by qRT-PCR using FFPE samples accurately recapitulates the prognostic performance of the GGI. Moreover the PCR-GGI was conducted with success from 10 years old FFPE samples. Fourteen percent of the samples have been excluded from the study and this number is expected to be lower using recent FFPE samples.

Additionally, as illustrated in this manuscript, the PCR-GGI has several advantages which highlight its clinical relevance.

First, as illustrated previously for the GGI, the PCR-GGI is able to identify high and low-risk patients within the subgroup of patients with intermediate histological grade tumors, possibly improving treatment decision making for these patients.

Second, as the GGI, the PCR-GGI is able to identify a higher proportion of early breast cancer patients at low risk of recurrence than do the clinical guidelines. This means that the number of patients that would receive unnecessary treatment could be reduced by applying this molecular assay.

Third, the PCR-GGI is not subject to reproducibility and inter-observer variability problems such as the histological grading. Moreover, the PCR-GGI's procedure developed at Jules Bordet was easily transferred at the Josephine Nefkens Institute, Rotterdam following some inter-laboratory tests which gave great confidence in the reproducibility of the assessment of the PCR-GGI. Also, the PCR-GGI cut-off for positivity identified at the Jules Bordet Institute was easily applied on the Josephine Nefkens Institute assays.

Fourth, the PCR-GGI can classify ER-positive BC patients treated either with adjuvant tamoxifen only or first-line tamoxifen for advanced disease into clinically relevant subgroups. We first observed a statistically significant association between a high PCR-GGI score and a higher risk of recurrence in ER-positive BC samples from patients that received adjuvant tamoxifen for primary disease across two different BC populations. We also observed a statistically significant association between a high PCR-GGI score and a higher risk of progression in ER+ BC samples originated from patients that received first-line tamoxifen for advanced disease. However, the determination of ER positivity might also play a role in the assessment of the performance of the PCR-GGI since we report here a discordance of 25% between IHC and RT-PCR assessment of ER.

Finally, the PCR-GGI has two practical advantages: 1/ it requires only very small amounts of routinely available FFPE samples, whereas the original GGI requires fresh-frozen material, and 2/ it allows the use of a SYBR-based technology, instead of the specific Taqman probes (Applied Biosystems), providing the advantages of being easy to use and cheaper than specific probes. Therefore, we might envisage that this test could be carried out in local certified pathology laboratories.

Additionally, several studies have demonstrated that tumors with characteristics associated with poor histological grade and high proliferating index tended to respond better to chemotherapy [13,14]. As our signature was derived from the histological grade we might then hypothesize that the PCR-GGI not only quantifies the likelihood of BC recurrence in women with node-negative ER+ BC, it might also predict the magnitude of chemotherapy benefit.

## Conclusion

As for the GGI, the clinical potential of the PCR-GGI is obvious since: 1/ the three category of histological grading is replaced by a two-category grading clinically more relevant, 2/ it classifies ER-positive BC patients into two clinically relevant subgroups, and 3/ the genes expression score is not subject to inter-observer variability as is the histological grade. Additionally, the PCR-GGI presents the advantage that it can be evaluated on FFPE samples, which are more widely available than frozen samples.

## Methods

### Patients and Samples Collection

A small series of 19 primary BC from which frozen, FFPE tissues and their corresponding microarray data were available, further referred as "IJBtest", was used to compare the accuracy and the concordance of the microarray-derived GGI to the qRT-PCR genomic grade index (PCR-GGI).

The prognostic value of the PCR-GGI was evaluated in a consecutive series of 212 FFPE primary BC samples collected at the Jules Bordet Institute from 1995 to 1996, referred as "IJB95/96". Median follow-up was of 7 years.

The clinical value of the PCR-GGI for tamoxifen benefit was evaluated in three independent BC populations derived from two independent institutions:

1) A series of 77 frozen ER+, node-negative and -positive adjuvant tamoxifen-only treated primary BC collected at the John Radcliff Hospital (Oxford, UK) for which microarray data were available, referred as "OXFD". Median follow-up was of 5 years.

2) A series of 139 frozen ER+, node positive, adjuvant tamoxifen only treated primary BC collected from 1979 to 2001 at the Josephine Nefkens Institute of the Erasmus MC Rotterdam (NL), referred as "JNIadj". Median follow-up was of 3.5 years.

3) A series of 270 frozen ER+ BC samples from hormone-naïve patients that received first-line tamoxifen for metastatic disease [of which 52 patients received adjuvant chemotherapy], collected from 1979 to 2001 at the Josephine Nefkens Institute of the Erasmus MC Rotterdam, (NL), referred as "JNImeta". Median follow-up was of 5 years.

All frozen as well as FFPE samples contained >30% of invasive tumor cells. This study was approved by the ethics committee of the Institut Jules Bordet, the John Radcliffe Hospital and the Josephine Nefkens Institute of the Erasmus MC Rotterdam (MEC 02.953). The JNIadj and JNImeta samples involved coded tumor tissues and execution of this part of the study was performed in accordance with the Code of Conduct of the Federation of Medical Scientific Societies in the Netherlands .

### RNA extraction and purification

RNA from frozen samples was extracted for the OXFD and IJBtest cohorts from four 10-μm sections using Trizol reagent according to the supplier's instructions (*Invitrogen Life Technologies, Carlsbad, CA*). For the JNIadj and JNImeta cohorts RNA was extracted from 30-μm sections using RNABee (Campro Scientific) as previously described [15].

RNA from FFPE samples was extracted from three 10-μm sections using the MasterPure Purification kit (*Epicentre, Madison, WI*) after paraffin removal with xylene. A DNase I treatment step was included.

To evaluate RNA quality and concentration, RNA from frozen samples were loaded onto an Agilent RNA 6000 NanoLabChip (RNA LabChip, Agilent Technology, Santa Clara, CA) in combination with the Bioanalyser 2100. Samples with a total area under the 28S and 18S bands of less than 15% of the total RNA band area, as well as a 28S/18S ratio of less than 1.1, were considered to be degraded. The RNA concentration from FFPE samples was analyzed with the NanoDrop^® ^ND-1000 UV-Vis Spectrophotometer (NanoDrop Technologies, Wilmington, DE, USA).

### cDNA synthesis

For the "OXFD" and "IJB95/96" cohorts reverse transcription (RT) was performed using a Super-Script First-Strand Synthesis kit for RT-PCR (Invitrogen Corp., Carlsbad, CA). Total RNA (300 ng) was reverse transcribed in a final volume of 21 μl with 50 ng of random hexamers. For the "JNIadj" and "JNImeta" cohorts cDNA was synthesized from 2 μg total RNA using random hexamers and oligo dTs, as previously described [15]. A RNase H treatment step was included in all protocols.

### PCR amplification

Quantitative PCR (qPCR) reactions were performed in 96-well plates using Applied Biosystems Prism 7900 HT (TaqMan instruments) and a Stratagene Mx3000P QPCR System (Agilent Technologies, Waldbronn, Germany). Gene expressions were measured in duplicate using 5 ng equivalent cDNA per reaction well. Amplifications were performed in 25 μl PCR mixture containing 300-600 nM of each primer and 12,5 μl 2× SYBR Green PCR Master Mix (Applied Biosystems). After 2 min at 50°C and 10 min at 95°C, cDNA was subjected to 40 cycles of PCR with a denaturation step at 95°C for 30 sec followed by a combined annealing/extension step at 60°C for 1 min.

Primers of the selected genes were designed using the Primer Express software (PE Applied Biosystems-see Table 4 for primer sequences).

**Table 4:** Forward and reverse primers sequences for signature and normalization genes.

**Gene Name**	**Accession number**	**Primer sequence**
**4-genes signature**		

*myb myeloblastosis viral oncogene homolog (avian)-like 2 (MYBL2)*	NM_002466	-AGCAAGTGCAAGGTCAAATGG -CTGTCCAAACTGCCTCACCA

*karyopherin alpha 2 (KPNA2)*	NM_002266	-TAAGGCAGATTTTAAGACACAAAAGG -GTTCAACTGTTCCACCACTGGTATA

*cell division cycle 2 (CDC2)*	NM_001786	-GCCGCCGCGGAATAAT -CCTTCTCCAATTTTCTCTATTTTGGT

*cell division cycle 20 homolog (CDC20)*	NM_001255	-CTTCCCTGCCAGACCGTATC -CCAATCCACAAGGTTCAGGTAATA

**Genes of Normalization**		

*glucuronidase beta (GUS)*	NM_000181	-GAGTGGTGCTGAGGATTGGC -TCTAGCGTGTCGACCCCATT

*TATA box binding protein (TBP)*	NM_003194	-GCCCGAAACGCCGAATAT -TCGTGGCTCTCTTATCCTCATGA

*ribosomal protein, large, P0 (RPLP0)*	NM_001002	-ACCAAGGAGGACCTCACTGAG -ACCAGCACGGGCAGCAG

*transferrin receptor (TFRC)*	NM_003234	-GGAGCCAGGAGAGGACTTCC -TTCTCCGACAACTTTCTCTTCAGG

**ER**		

*estrogen receptor 1 (ESR1)*	NM_000125	-TGTTCCAAACCCATCGTCAGT -GCACCTGCTCATGGGACAA

*:UK = Unknown.

Four housekeeping genes were selected on the basis of the literature as reference genes for data normalization: *TFRC, GUS, RPLPO *and *TBP *[16]. All PCR assays were run in duplicate. For all our samples the average value for the housekeeping gene is highly similar therefore all samples with no expression or a lower Ct value of the housekeeping genes compared to the value of the global population were excluded from the analyses. Six percent of frozen and 14% of FFPE samples were then excluded from the analyses.

PCR-GGI assay was developed at the Jules Bordet Institute on Jules Bordet populations and validated both at the Jules Bordet (IJB95/96, OXFD) and the Josephine Nefkens Institutes (JNIadj, JNImeta) on independent populations.

## Data analysis

### qRT-PCR data normalization and PCR-GGI computation

The average of the housekeeper genes values was used as reference and a Ct value of GGI genes was defined for each sample by taking off this average. To calculate the reduced gene expression grade index, we averaged the normalized values of the four GGI genes. This is illustrated by the following formula:



where s_*j *_is the PCR-GGI score for patient *j*, G_*i *_is the raw gene expression of the *i*^th ^gene in the PCR-GGI signature for patient *j*, and  is the mean of the housekeeping genes used for normalization.

In order to classify patients into low- and high-risk groups, a cut-off was defined as the middle point between the averages of the values of histological grade 1 and histological grade 3 tumors. This cut-off was defined on frozen samples (OXFD). To correct for the difference in scale of PCR-GGI scores between frozen and FFPE samples, we transformed the qRT-PCR scores derived from FFPE ER-positive patients such that the mean and standard deviation were equal to those from frozen samples:



where s_*j *_is the PCR-GGI score for patient *j *from a FFPE sample, μ_*f *_and μ_*p *_are the mean value of the PCR-GGI scores for ER-positive frozen and FFPE tumor samples respectively, and σ_*f *_and σ_*p *_are the standard deviation of the PCR-GGI scores of ER-positive frozen and FFPE tumor samples respectively.

### Evaluation of estrogen receptor (ER) and Ki-67 expression by immunohistochemistry (IHC)

Paraffin-embedded blocks routinely fixed in neutral buffered formalin were cut on poly-L-Lysine-coated slides and stained with antibodies to ER clone 6F11 (dilution 1/40, Novocastra, Newcastle, UK) and to Ki-67 clone MIB-1 (dilution 1/50; DAKO, Carpinteria, CA). Antigen retrieval was performed in citrate buffer pH6 as previously described [17]. The Ventana Nexes automated immunostainer (Ventana Medical Systems, Tucson, Az) was used with standard reagents. Tumors were defined as ER negative if < 10% of tumor cells had positive immunostaining and as highly proliferating samples if >15% of tumors cells had positive Ki-67 immunostaining.

### ER evaluation by qRT-PCR

In order to consistently identify the ER status of BC patients, we clustered the tumors using the *ESR1 *qRT-PCR expressions by fitting Gaussian mixture models [18] with equal and diagonal variance for all clusters. We used the Bayesian Information Criterion [19] to test the number of components. Each tumor was then automatically classified as ER-positive or ER-negative using the maximum posterior probability of membership to these two clusters.

### Survival analysis

We considered DMFS of BC patients as endpoint for the "IJB95/96" and "JNIadj" data sets and PFS as endpoint for the "JNImeta" data set. Criteria for follow up, type of response and response to therapy were defined by standard International Union Against Cancer criteria of tumor response [20]. PFS results were described previously [21]. Survival curves were computed using Kaplan-Meier product limit estimator. Hazard ratios for continuous and discrete variables were estimated through Cox's proportional hazard regression models. A backward stepwise selection based on likelihood ratio tests was used for the identification of the covariates to be kept in the final Cox regression models.

All p-values were two-tailed and p-values < 0.05 were considered statistically significant. All statistical analyses were carried out using R version 2.5.1 and SPSS version 15 (SPSS Inc. 1999, Chicago IL.).

## List of abbreviations

**BC**: breast cancer; **cDNA**: complementary deoxyribonucleic acid; **Ct**: treshold cycle; **DMFS**: distant metastasis free survival; **ER**: estrogen receptor; **FFPE**: formalin fixed paraffin embedded; **GGI**: genomic grade index; **HR**: hazard ratio; **IHC**: immunohistochemistry; **IJB95/96**: series of 212 FFPE primary BC sample collected at Jules Bordet Institute from 1995 to 1996; **IJBtest**: series of 19 primary BC from which frozen, FFPE tissues and corresponding microarray data were available; **JNIadj**: series of 139 frozen ER+, node positive, adjuvant tamoxifen treated primary BC collected from 1979 to 2001 at the Josephine Nefkens Institute of the Erasmus MC Rotterdam; **JNImeta**: series of 270 frozen ER+ BC from hormone-naïve patients that received first-line tamoxifen for metastatic disease, collected from 1979 to 2001 at the Josephine Nefkens Institute of the Erasmus MC Rotterdam; **MPF**: M-phase promoting factor; **OR**: objective response; **OXFD**: series of 77 frozen ER+ adjuvant tamoxifen treated primary BC collected at the John Radcliff Hospital for which microarray data were available; **PCR-GGI**: genomic grade index converts to a qRT-PCR assay; **PFS**: progression free survival; **qRT-PCR**: real-time reverse transcription polymerase chain reaction; **RNA**: ribonucleic acid.

## Competing interests

CS is named inventor on a patent application for the Genomic Grade signature used in this study. There are no other conflicts of interest.

## Authors' contributions

*JT *has made contributions to method conception and design, acquisition of expression data, analysis and interpretation of data. He has been involved in drafting the manuscript and given final approval of the version to be published. *AMS *has made contributions to acquisition of expression data, analysis, interpretation of data and in drafting the manuscript. *BHK and CD *have contributed to statistical analyses, contributed to method design and in drafting the manuscript.*GR and DL *have been involved in the acquisition of data. *VD *has been involved in method design, acquisition of data, statistical analyses and in drafting the manuscript. *ALH, MP, JAF and CS*: have been involved in method design and in drafting the manuscript. All authors read and approved the final manuscript.
